# Classification, diagnosis and potential mechanisms in Pontocerebellar Hypoplasia

**DOI:** 10.1186/1750-1172-6-50

**Published:** 2011-07-12

**Authors:** Yasmin Namavar, Peter G Barth, Bwee Tien Poll-The, Frank Baas

**Affiliations:** 1 Department of Genome Analysis, Academic Medical Center, University of Amsterdam, Meibergdreef 9, 1105 AZ Amsterdam, The Netherlands; 2 Division of Pediatric Neurology, Emma's Childrens Hospital, Academic Medical Center, University of Amsterdam, Meibergdreef 9, 1105 AZ Amsterdam, The Netherlands

## Abstract

Pontocerebellar Hypoplasia (PCH) is group of very rare, inherited progressive neurodegenerative disorders with prenatal onset. Up to now seven different subtypes have been reported (PCH1-7). The incidence of each subtype is unknown. All subtypes share common characteristics, including hypoplasia/atrophy of cerebellum and pons, progressive microcephaly, and variable cerebral involvement. Patients have severe cognitive and motor handicaps and seizures are often reported. Treatment is only symptomatic and prognosis is poor, as most patients die during infancy or childhood. The genetic basis of different subtypes has been elucidated, which makes prenatal testing possible in families with mutations. Mutations in three tRNA splicing endonuclease subunit genes were found to be responsible for PCH2, PCH4 and PCH5. Mutations in the nuclear encoded mitochondrial arginyl- tRNA synthetase gene underlie PCH6. The tRNA splicing endonuclease, the mitochondrial arginyl- tRNA synthetase and the vaccinia related kinase1 are mutated in the minority of PCH1 cases. These genes are involved in essential processes in protein synthesis in general and tRNA processing in particular. In this review we describe the neuroradiological, neuropathological, clinical and genetic features of the different PCH subtypes and we report on *in vitro *and *in vivo *studies on the tRNA splicing endonuclease and mitochondrial arginyl-tRNA synthetase and discuss their relation to pontocerebellar hypoplasia.

## Review

### Pontocerebellar Hypoplasias

**Figure 1:**
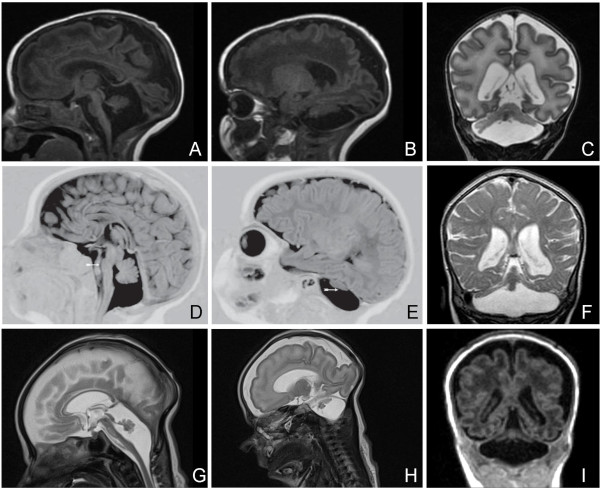
**MRI sections of cases with PCH type 1, type 2 and type 4**. The images of the PCH1 case were kindly provided by Professor Darin, The Queen Silvia. Children's Hospital, Gothenburg University, Sweden. **1A-C**: Images of a 2 wk old neonate with PCH1. **1A: **Mid-sagittal section (T1) shows vermal hypoplasia and marked cerebellar hypoplasia. **1B: **Lateral sagittal section (T1) shows severe hypoplasia of the cerebellar hemispheres. **1C: **Coronal section (T2) shows flattened cerebellar hemispheres which also display some atrophy. The vermis is relatively spared. **1D-E**: Images of a 2 months old baby with PCH2. **1D: **Mid-sagittal section (T1IR) shows a flat ventral pons and vermal hypoplasia. **1E: **Lateral sagittal section (T1IR) shows severely hypoplastic cerebellar hemispheres (arrow) leaving most of the posterior fossa empty. **1F: **Coronal section (T2) of a 9 months old infant with PCH2 shows flat cerebellar hemispheres and mild vermal hypoplasia (dragonfly configuration). Cerebral cortical atrophy is also present. **1G-I: **Images of a 31+5 weeks neonate with PCH4. **1G: **Mid-sagittal section (T2) shows severe vermal hypoplasia and ventral pontine flattening. **1H: **Lateral sagittal section (T2) shows severe hypoplasia of the cerebellar hemispheres. Above the tentorium there is an increased distance between the cortical surface and the skull visible, which is probably due to diminished brain growth in utero. **1I: **Coronal section (T1) shows extremely small and flattened cerebellar hemispheres and severe vermal hypoplasia. Immaturity of cerebral cortex and enlarged ventricles are also visible.

The name Pontocerebellar Hypoplasia (PCH) originates from a report of Brun almost a century ago, in which he described human brain development and abnormalities associated with brain development. Cerebellar Hypoplasia is described as dwarfed growth of the cerebellum [1]. Seven years later Brouwer suggested that pontocerebellar hypoplasia is possibly due to degeneration rather than to hypoplasia [2]. Subsequent reports described the pathology as atrophy of cerebellar hemispheres with relative sparing of the flocculi and vermis and apparent fragmentation of the cerebellar dentate nucleus [3-5]. The first reported case of PCH which included specific clinical details was probably by Krause [4]. He reported a child with swallowing problems, spasticity and complete absence of cognitive and voluntary motor development with the pathological profile of PCH. In retrospect this may have been the first documented case on PCH type 2. Pfeiffer and Pfeiffer first reported the extrapyramidal component [5-8]. Barth *et al*. described a cluster of related families with PCH from a genetic isolate in the Netherlands as an inherited syndrome of microcephaly, dyskinesia and pontocerebellar hypoplasia [9]. A first attempt for classification was based on two subtypes; this divided PCH in cases with accompanying spinal anterior horn disease (type 1) and cases with chorea/dyskinesia (type 2) [10]. This classification was extended into five subtypes in 2006 and in 2007 a sixth was added [11,12]. The latest subtype that may be classified as PCH7, has been recently added to this list [13]. PCH now includes seven (PCH1-7) disorders. In most cases, especially in PCH1, PCH2, PCH4 and PCH5 prenatal onset of structural decline is well documented. In some milder cases cerebellar images suggest a perinatal or early postnatal onset. The clinical diagnosis is made on neuroradiological, neuropathological and neurological findings [14-18]. Neuroradiological findings in all subtypes are pontocerebellar hypoplasia and atrophy of ventral pons, cerebellum and to a lesser extent also the cerebral cortex (Figure 1). Neuropathologically, there is segmental degeneration of the cerebellar cortex with loss of Purkinje cells, fragmentation of the dentate nucleus and degeneration with neuronal loss and decreased folding of the inferior olivary nuclei in PCH1, PCH2, PCH4 and PCH5. Neuropathological studies in the other types are scarce or absent. Cerebellar hemispheres are usually more severely affected than the vermis and there is progressive loss of the ventral nuclei and transverse fibers in the pons [19]. Furthermore there is severe progressive microcephaly and variable ventriculomegaly. Severe intellectual deficit, swallowing problems and seizures are clinical features of all subtypes [9,18,20].

### Clinical features of the PCH subtypes

#### PCH1

PCH type 1 (Table 1) (PCH1, previously known as Norman's disease, ORPHA2254, MIM 607596) is characterized by pontocerebellar hypoplasia with additional loss of motor neurons in the anterior horn of the spinal cord, pathologically similar to the spinal muscular atrophies (SMA) [6,7]. Magnetic resonance imaging (MRI) of PCH1 patients always shows cerebellar hypoplasia in which the cerebellar hemispheres are variably affected; in some cases more flat and in other cases more preserved (Figure 1A-C). There is variable involvement of pons and cerebrum [21]. Patients suffer from severe hypotonia, pareses, central visual failure, dysphagia, respiratory insufficiency, psychomotor retardation and they usually die within the first year. The majority of patients also exhibits prenatal onset of symptoms such as congenital contractures and polyhydramnios. Microcephaly in most reported cases is not present at birth, but develops postnatally [7,10,21-25].

**Table 1:** PCH subtypes.

PCH	Clinical features	Pathological features	Gene(s)	**Ref**.
PCH1	Neonatal period: Hypotonia, impaired swallowing, congenital contractures and/or polyhydramnios, primary hypoventilation, progressive microcephaly. MRI: Pontocerebellar hypoplasia.	Cerebellar hypoplasia: hemispheres > > vermis, areas of stunted or absent folial development. Cerebellar dentate nucleus present as tiny remnants. Olivary nucleus: absent folding and gliosis. Pons: loss of ventral nuclei and transverse fibers. Spinal cord: Anterior horn cell degeneration. Peripheral nerves and muscle: chronic denervation.	One family with the common *TSEN54 *mutation. One case with missense plus splice site mutations in *RARS2*. One atypical mild family with nonsense mutations in *VRK1*. Locus unknown in the majority.	[6,7,10,20,21,40,42]

PCH2	Neonatal period: Clonus, impaired swallowing. Infancy and later: Chorea, variable spastic pareses; progressive microcephaly. Severe impairment of cognitive and motor development. MRI: Variable neocortical atrophy, pontocerebellar hypoplasia.	Cerebellar hypoplasia: hemispheres > > vermis. Segmental degeneration of cerebellar cortex. Fragmentation of cerebellar dentate nucleus. Olivary nucleus: neuron loss and decreased folding. Pons: progressive loss of ventral nuclei and transverse fibers. Cerebral cortex: progressive atrophy	*TSEN54: *p.A307S, A307S most common in 75 families. Rarely: Other *TSEN54 *missense mutations in 3 families, *TSEN2 *mutations in 2 families, *TSEN34 *mutations in 1 family.	[15,18-20,28,37]

PCH3	Neonatal period: Hypotonia, impaired swallowing. Infancy and later: Short stature progressive microcephaly, optic atrophy. MRI: Neocortical and cerebellar atrophy, pontocerebellar hypoplasia; Pons and cerebellum equally affected.	No autopsies performed.	Locus on chromosome 7 q in 2 families.	[30-32]

PCH4	Neonatal period: Hypertonia, severe clonus, polyhydramnios and/or contractures; primary hypoventilation. MRI: Delayed neocortical maturation, pontocerebellar hypoplasia; microcephaly on autopsy.	Cerebellar hypoplasia: hemispheres > > vermis, areas of stunted or absent folial development. Cerebellar dentate nucleus present as tiny remnants Olivary nucleus: absent folding and gliosis. Pons: loss of ventral nuclei and transverse fibers.	*TSEN54: *Compound heterozygosity for p.A307S plus nonsense or splice site mutations in 10 families. Three missense mutations in 1 family.	[14,16,19,20,37]

PCH5	Prenatal/neonatal period: Clonus or seizures. Neonatal period: Persistent clonus, microcephaly and pontocerebellar hypoplasia on autopsy.	Cerebellar hypoplasia: cortical involvement as in PCH4, but vermal cortex more extensively affected than hemispheric cortex; subtotal loss of cerebellar dentate nucleus. Olivary nucleus: absent folding. Pons: loss of ventral nuclei and transverse fibers.	*TSEN54*: Compound heterozygosity for p.A307S plus splice site mutation in 1 family.	[11,20]

PCH6	Neonatal period: Hypotonia, clonus, impaired swallowing. Infancy and later: Progressive microcephaly, spasticity, elevated CSF lactate, edema of extremities. MRI: Neocortical and cerebellar atrophy, pontocerebellar hypoplasia; Pons and cerebellum equally affected.	No autopsies performed.	Missense and splice site mutations in *RARS2 *in 2 families.	[12,39]

PCH7	Neonatal period: No palpable gonads with a micropenis. Hypotonia. Infancy and later: Regression of penis. Progressive microcephaly, seizures, respiratory distress, poor feeding. MRI: Pontocerebellar hypoplasia.	Cerebellar hypoplasia: absence of cerebellar hemispheres with neuronal loss. Olivary nucleus: absent. Pons: loss of ventral nuclei and transverse fibers. Cerebral cortex: progressive atrophy.	No locus.	[13]

Adapted and extended from Namavar *et al*. [38].

#### PCH2

PCH type 2 (PCH2, ORPHA2524, MIM 277470, 612389, 612390) is the most frequently reported and therefore the best studied subtype (Table 1) [10,18-20,26]. So far at least 81 families have been reported [20,27,28]. Extrapyramidal dyskinesias and dystonia are the major features of PCH2, pure spasticity is reported in a minority. Other clinical features include impairment of swallowing from birth on, jitteriness in the neonatal period, central visual failure and seizures. There is no involvement of the spinal anterior horn cells in any of the cases that have been studied post-mortem. Life expectancy is unpredictable, as age of death ranges from the neonatal period to well into adulthood; most patients however do not reach puberty [20]. Recently chances for survival have become greater due to improved care, such as tube feeding and gastrostomy. Typical brain MRI findings are a dragonfly-like cerebellar pattern on coronal sections, in which the cerebellar hemispheres are flat and severely reduced in size and the vermis is relatively spared (Figure 1D, E, F) [20]. Mild or severe atrophy of the cerebral cortex is observed in 40% of the PCH2 cases. On MRI myelination is delayed, but demyelination has not been observed [20].

Prenatal diagnosis by ultrasound imaging is not yet possible in the second trimester of pregnancy, therefore molecular genetic testing is required for prenatal diagnosis in high risk pregnancies [29].

#### PCH3

PCH type 3 (PCH3, ORPHA97249, MIM 608027) also known as cerebellar atrophy with progressive microcephaly (CLAM), is a very rare subtype of PCH (Table 1). Only three families have been described so far. All cases had short stature, seizures, hypotonia and in four of the five cases optic atrophy was reported [30-32]. In the most recent reported case, the patient suffered from an additional severe Vitamin A deficiency with unknown cause [32].

#### PCH4

PCH type 4 (PCH4, olivopontocerebellar hypoplasia (OPCH), ORPHA166063, MIM 225753) shares similarities with PCH2; however PCH4 is more rare and the disease course is more severe (Table 1). Up to now 18 families have been reported with a PCH4 phenotype [14,16,20,28,33-36]. Patients exhibit more severe perinatal symptoms such as excessive, prolonged general clonus, congenital contractures, polyhydramnios and primary hypoventilation the latter necessitating prolonged mechanical ventilation. Weaning from ventilatory support is often impossible making survival through the neonatal period exceptional [20]. The pathology seen in PCH4 deviates in some regards from PCH2. A striking pathological distinction in PCH4 is the C-shaped form of the inferior olives and large denuded areas without folia in the cerebellar hemispheric cortex, both phenomena suggesting an early prenatal onset [14,19,37]. Other striking features in PCH4 are pericerebral cerebrospinal fluid (CSF) accumulation, wide midline cava and delayed neocortical maturation; all suggesting prenatal decline of cerebral growth. Additionally the cerebellar vermis is more severely affected (Figure 1G, H, I) [20,38]. MRI analysis is therefore helpful in the clinical diagnosis of PCH4.

#### PCH5

Only one family with PCH type 5 (PCH5, ORPHA166068, MIM 610204) has been described (Table 1). In this subtype of PCH, patients displayed fetal onset of seizure-like activity in combination with severe olivopontocerebellar hypoplasia and a severely affected cerebellar vermis [11]. Autopsy of the three published siblings showed diffuse brain volume loss, C-shaped inferior olivary nuclei, absent or immature dentate nuclei and cell death which was more pronounced in the cerebellar vermis than in the hemispheres. No evidence was found for spinal cord involvement.

In retrospect there is an arbitrary difference between PCH4 and PCH5 [38]. In PCH5 the vermis was more affected than the hemispheres, whereas in PCH4 the vermis and the hemispheres are both severely affected, with the emphasis on the hemispheres. The prenatal seizure-like activity observed in PCH5 appears similar to the severe neonatal clonus observed in PCH4 [16]. The primary hypoventilation observed in PCH5 is also a typical aspect of PCH4 [20].

#### PCH6

PCH type 6 (PCH6, ORPHA166073, MIM 611523) is a rare subtype of PCH (Table 1). The first published family with PCH6 is a Sephardic Jewish family with three siblings exhibiting cerebellar and vermal hypoplasia, infantile encephalopathy, dysphagia, seizures, progressive microcephaly and generalized hypotonia followed by spasticity [12]. No developmental milestones were reached. Biochemical investigation of mitochondrial complexes showed reduced activity of mitochondrial complexes I, III, and IV in muscle, while activity of complex II was normal. Elevated CSF lactate levels were found. Another case by Rankin *et al*. with a PCH6 phenotype in combination with progressive encephalopathy and edema, was suggestive of PEHO syndrome (Progressive Encephalopathy with Oedema, Hypsarrhythmia and Optic atrophy) [39]. No autopsies have been performed in PCH6 cases so far.

#### PCH7

A new subtype was proposed based on a profile combining genital abnormalities in combination with PCH. We tentatively classify this as PCH7 (Table 1). The male patient had impalpable testes with a micropenis at birth and an XY karyotype. In the following weeks he developed progressive microcephaly, swallowing problems, hypotonia, respiratory distress, absent tracking movements, a head lag and seizures. MRI at the age of 16 weeks showed pontocerebellar hypoplasia and cerebral atrophy. At 19 weeks of age, regression of penile corporeal tissue was noted. He died at 5 1/2 months of age [13].

### Genetics of PCH

#### TSEN-related PCH and genotype-phenotype correlations

Through homozygosity mapping in a cluster of related families with PCH2, the genetic basis for PCH2 was identified [37]. All patients were homozygous mutant for an amino acid change of an alanine into a serine at position 307 (p.A307S, common mutation) in the transferRNA (tRNA) splicing endonuclease subunit 54 gene (*TSEN54*) (Table 2). Ninety percent of the well-defined PCH2 cases carried this mutation [37]. This *TSEN54 *mutation correlated strongly with jitteriness, clonus, dyskinesia and/or dystonia and with flat cerebellar hemispheres on coronal MRI compared to those PCH cases where no mutation was identified (Figure 1F) [20].

**Table 2:** Pathogenic mutations in PCH.

Gene	Nucleotide position	Protein position	Subtype
TSEN54	c.178_215del	p.E60AfsX109	PCH4
TSEN54	c.285G > C	p.A95A Splice site mutation	PCH4
TSEN54	c.277T > C	p.S93P	PCH4
TSEN54	c.371G > T	p.G124V	PCH2
TSEN54	c.370-2A > G	p.G124_Q138del	PCH4
TSEN54	c.468+2T > C	Splice site mutation	PCH5
TSEN54	c.736C > T	p.Q246X	PCH4
TSEN54	c.919G > T	p.A307S (common)	PCH1, PCH2, PCH4, PCH5
TSEN54	c.953delC	p.P318QfsX23	PCH4
TSEN54	c.1027C > T	p.Q343X	PCH4
TSEN54	c.1056_1057del	p.R353GfsX81	PCH4
TSEN54	c.1170_1183del	p. V390fsX39	PCH4
TSEN54	c.1251A > G	p.P417P Splice site mutation	PCH4
TSEN54	c.1430+2T > A	Splice site mutation	PCH4
TSEN54	c.1537T > G	p.Y513D	PCH4
TSEN34	c.172C > T	p.R58W	PCH2
TSEN2	c.926A > G	p.Y309C	PCH2
TSEN2	c.960+1delGTAAG	Splice site mutation	PCH2
RARS2	c.35A > G	p.Q12R	PCH1, PCH6
RARS2	c.110+5A > G	Splice site mutation	PCH1, PCH6
RARS2	c.1024A > G	p.M342V	PCH6
VRK1	c.1072C > T	p.R358X	PCH1

Note that in PCH4 and PCH5 there is compound heterozygosity for a nonsense or a splice site mutation plus a missense mutation (p.A307S, *TSEN54*).

In rare occasions, mutations are found in two of the three other subunit genes of the tRNA splicing endonuclease, *TSEN2 *and *TSEN34 *(Table 2) [20,37]. Four patients have been described so far; three with *TSEN2 *mutations and one with a *TSEN34 *mutation [20]. These cases have a PCH2 phenotype, as they exhibited spasticity and/or dyskinesias. Other missense mutations than the p.A307S in *TSEN54*, have been associated with PCH2 as well (Table 2). Some of these patients with a rare mutation in *TSEN54 *and the patient with a *TSEN34 *mutation have relatively mild involvement of pons and cerebellum. On early coronal MRI the cerebellar hemispheres are not completely flat, but fill the posterior fossa almost completely, suggestive of postnatal atrophy rather than hypoplasia [20]. Because only a few patients with *TSEN2*, *TSEN34 *and rare *TSEN54 *missense mutations other than p.A307S have been diagnosed thus far, one should be cautious with generalizations about their phenotypes.

Whereas missense mutations in *TSEN54 *underlie PCH2, heterozygous missense- plus heterozygous nonsense or splice site mutations in *TSEN54 *underlie the more severe PCH4 (Table 1 Table 2) [20,27,37]. In one case, three *TSEN54 *missense mutations were found in PCH4. This case was homozygous mutant for the common mutation, plus another missense mutation (p.S93P) on one of the alleles, giving rise to a PCH4 phenotype. Nonsense and splice site mutations in *TSEN54 *are associated with increased severity of hypoplasia of pons and cerebellum and immaturity of the cerebral cortex with more perinatal symptoms and an earlier lethality than seen in PCH2.

As in PCH4, a heterozygous missense mutation (p.A307S) plus a heterozygous splice site mutation (c.468+2T > C) in *TSEN54 *has been found to be responsible for PCH5 (Table 2) [38]. Although milder, the clinical findings in PCH2 are similar to PCH4 and PCH5. Therefore PCH5, PCH4 and PCH2 represent a spectrum of clinical manifestations caused by different mutations in the *TSEN *genes (Table 1). It is still unclear whether PCH1 is part of this spectrum too, as the common mutation in *TSEN54 *was identified in one case from a family with three siblings with a PCH1 phenotype [40]. DNA was only available in one of the three siblings. Post-mortem examination revealed neuronal cell loss of the anterior horns of the cervical cord [40]. PCH1 seems to be more heterogeneous than PCH2/PCH4 and several genes are already involved in the minority of the PCH1 cases.

Reliable estimations of the incidence of the common/p.A307S mutation (*TSEN54*) are difficult to obtain. Although PCH2 with this underlying mutation is the most common form of PCH, it is still a rare disease and clusters in isolated communities. In the Dutch and German population the carrier frequency of the common mutation is 0.004 [37]. With 184 915 newborns in the Netherlands in the year 2009 one would expect 3 affected children per annum [41]. However, since the p.A307S mutation occurs in closed communities, where consanguinity occurs, probably more affected children are born per year, than one would expect based on carrier frequency in unrelated Caucasian individuals. Therefore preconceptional testing with prenatal diagnosis for this disease in selected regions is advised.

#### RARS2-related PCH

Following homozygosity mapping in the first published PCH6 family, a homozygous intronic splice site mutation (c.110+5A > G) was found in the gene for the nuclear encoded mitochondrial arginyl-tRNA synthetase (*RARS2*) (Table 2) [12]. A second PCH6 case with additional progressive encephalopathy and edema was compound heterozygote for *RARS2 *missense mutations (Table 2) [39].

Mutations in *RARS2 *were identified in one case with a PCH1 phenotype [20]. Although this case had high CSF lactate levels, which is normally not reported in PCH1, post-mortem examination revealed a neuropathological profile that fits a PCH1 phenotype with loss of spinal anterior horn neurons.

#### Other genes and loci involved in PCH

Nonsense mutations in the Vaccinia Related Kinase1 gene (*VRK1*) were reported to be associated with pontocerebellar hypoplasia plus SMA in one atypical PCH1 family of Ashkenazi Jewish origin [42]. Despite the severe microcephaly at birth (fronto-occipital circumference -3SD and -6SD), the three affected children were mildly delayed in their developmental milestones. The proband was e.g. able to walk at the age of 3 years; however she became wheelchair bound later in life and eventually died at the age of 11 years. Cognitive impairment was stated as mild mental retardation, whereas in typical PCH1 cases there is severe mental retardation and no developmental milestones will be achieved [7,10,21-25]. Up to now no other cases with *VRK1 *mutations have been reported.

Mutations or deletions in the survival motor neuron gene (*SMN1*) cause SMA. In PCH1 linkage to the *SMN *genes has been excluded; although the motor neuron loss observed in PCH1 is morphologically similar to the motor neuron loss in SMA [43,44]. There is no locus for the majority of the PCH1 cases and no other genes have been linked to PCH1 yet, with the exception of rare cases with *TSEN54, RARS2 *and *VRK1 *mutations (Table 2). Fifteen families with a PCH1 phenotype have been published thus far; only in 3 families mutations were identified [6,7,10,20-25,34,40,42,44-51]. Further research on these and other candidate genes in PCH1 is necessary to identify mutations involved in the remaining majority of the PCH1 cases.

Linkage on chromosome 7 q was found in two of the three families with PCH3, but no causative gene has been found [30,31]. Unfortunately no linkage analysis was performed in the most recently published case with PCH3 [32].

There is no locus yet for PCH7, however sequencing of the coding regions in *TSEN54*, *TSEN34*, *TSEN2*, *TSEN15 *and *RARS2 *yielded no mutations. FISH analysis of *SRY *and *Xq12 *and a CGH-array appeared to be normal, as well as ARX expansion analysis [13].

### Management and Treatment

There is no cure for PCH: Management is only symptomatic and includes nutritional support by percutaneous endoscopic gastrostomy (PEG feeding), treatment of dystonia, dyskinesias and seizures. Sometimes respiratory support is provided. The chorea in PCH2 is difficult to treat, but physiotherapy may ease cases with severe dystonia or spasticity. Levodopa treatment appeared beneficial in some cases [52].

Life-threatening complications of PCH are cot death, sleep apnea and malignant hyperthermia with rhabdomyolysis with extreme elevation of plasma creatine kinase. Sleep apnea can be detected by sleep monitoring. Malignant hyperthermia should be prevented by sufficient hydration and monitoring especially during periods of infection [26].

### Other diseases with (ponto)cerebellar hypoplasia

There are several other diseases that one may consider when a patient presents with pontocerebellar hypoplasia, see also Table 3 for an indication.

**Table 3:** Differential diagnostic options for PCH.

Differential diagnosis	Cerebellar Hypoplasia plus:	Gene(s)	Pathways involved	Key references
***Genetic diseases with cerebellar hypoplasia and/or atrophy and variable cerebral cortical atrophy***				
PCCA	Progressive Cerebello-cerebral atrophy, progressive microcephaly, spasticity, seizures, mental retardation and seizures.	Missense mutations in *SEPSECS*.	Selenocysteine synthesis	[53]
ICCA	Severe atrophy of cerebrum and cerebellum. Psychomotor retardation, clonus, seizures, spasticity, progressive microcephaly.	Missense mutations in *MED17*	Transcripition initiation	[54]
CDG type 1A and 1D	Hypotonia, ataxia, developmental delay, failure to thrive, microcephaly.	*PMM2 *(type1a), *ALG3 *(type 1d)	Glycoprotein biosynthesis	[55,56]
Phosphoserine aminotransferase deficiency	Low CSF concentrations of serine and glycine, seizures, progressive microcephaly, hypertonia and psychomotor retardation. White matter immaturity and cerebral atrophy.	*PSAT*	Serine biosynthesis	[59]
Different congenital mitochondrial disorders	Respiratory chain deficiencies plus several other abnormalities.	-	n/a	[60]
PEHO-syndrome	Progressive cerebellar atrophy, progressive encephalopathy, hypsarrythmie, edema and optic atrophy.	Unknown	Unknown	[62,63]

***Genetic diseases with cerebellar hypoplasia plus neocortical dysplasia***				

Dystroglanopathies: Walker-Warburg syndrome, MEB-disease, Fukuyama	Neocortical dysplasia. Mental retardation, eye abnormalities, seizures, impaired motor control.	*FKRP, LARGE, POMGNT1, POMT1, POMT2, FKTN*	Dystroglycan synthesis	[64,65]
Lissencephaly	Lissencephaly phenotype.	*RELN*	Neuronal migration	[67]
X-linked brain malformation phenotype with microcephaly and hypoplasia of the brainstem and cerebellum	Microcephaly, optic atrophy, sensorineural hearing loss, simplified gyri, developmental delay.	*CASK *	Neuronal migration; Part of MAGUK protein family, involved in signaling in both, pre- and post-synapses.	[66]
Congenital fibrosis of the extraocular muscles 3 with extraocular involvement	Ocular motility disorder, facial weakness, axonal peripheral neuropathy, delayed development, neocortical dysplasia and other neuronal migration disorders.	*TUBB3*	Neuronal migration	[68,69]

***Acquired cerebellar hypoplasia***				

Extreme prematurity (< 32 weeks)	Extreme prematurity.	n/a	n/a	[70]

#### Genetic diseases with cerebellar hypoplasia and/or atrophy and variable cerebral cortical atrophy

Recently a new PCH-like phenotype has been described: Progressive Cerebello-Cereberal Atrophy (PCCA). Patients with PCCA have postnatal atrophy of the cerebellar hemispheres, which is not typical for PCH, but some PCH patients do have this feature [20,30]. Patients with PCCA have progressive microcephaly, severe spasticity, mental retardation and in some cases seizures. Sequential brain MRI of patients shows the progressive nature of the cerebellar and cerebral atrophy. Missense mutations in the *O-*phosphoseryl-tRNA selenocysteine tRNA synthase gene (*SEPSECS*) are associated with this disease (Table 3) [53].

In infantile cerebral and cerebellar atrophy (ICCA) there is also cerebellar volume loss with psychomotor retardation, seizures, jitteriness, clonus, severe spasticity, visual problems, hypertonia and progressive postnatal microcephaly. Brain MRI shows severe atrophy of cerebrum and cerebellum (Table 3) [54].

Certain subtypes of the congenital disorders of glycosylation (CDG) disorders manifest with cerebellar hypoplasia as well. CDG-1a patients have generalized hypotonia, developmental delay, swallowing problems, failure to thrive and cerebellar hypoplasia, next to variable external dysmorphia and hematologic problems [55]. Cerebellar hypoplasia is also present in patients with CDG-1d, a rare form of CDG (Table 3) [56-58].

Phosphoserine aminotransferase deficiency is associated with low CSF concentrations of serine and glycine. Clinically patients exhibit seizures, progressive microcephaly, hypertonia and psychomotor retardation. MRI shows cerebral atrophy, poor white matter development and vermal hypoplasia. The authors did not mention the size and morphology of the cerebellar hemispheres (Table 3) [59]. Also in various pediatric mitochondrial disorders predominant cerebellar volume loss is relatively common, together with respiratory chain deficiencies (Table 3) [60,61]. Progressive cerebellar atrophy is commonly found in patients with PEHO-syndrome (Table 3) [62,63].

#### Genetic diseases with cerebellar hypoplasia plus neocortical dysplasia

Dystroglycanopathies cause neocortical dysplasia and variable pontocerebellar hypoplasia and microcephaly or hydrocephalus (Table 3) [64,65]. An X-linked brain malformation phenotype with a moderately simplified gyral pattern and mild cortical dysplasia, only visible on autopsy is due to mutations in the calcium/calmodulin dependent serine protein kinase gene (*CASK*). It can manifests with microcephaly, optic atrophy, sensorineural hearing loss and pontocerebellar hypoplasia (Table 3) [66]. Other diseases with cerebellar hypoplasia and additional neocortical dysplasia but easier to differentiate from PCH, are lissencephaly with agyria or very wide gyria and congenital fibrosis of the extraocular muscles type 3 (CFEOM3) with extraocular involvement like neocortical dysplasia and neuronal migration defects (Table 3) [67-69].

#### Acquired cerebellar hypoplasia

Extreme prematurity (< 32 weeks) in infants can also lead to cerebellar hypoplasia, due to disruption of normal brain development. One should be aware of this when a prematurely born neonate presents with these features (Table 3) [70,71].

### Pathogenesis

#### The function of the tRNA splicing endonuclease

**Figure 2:**
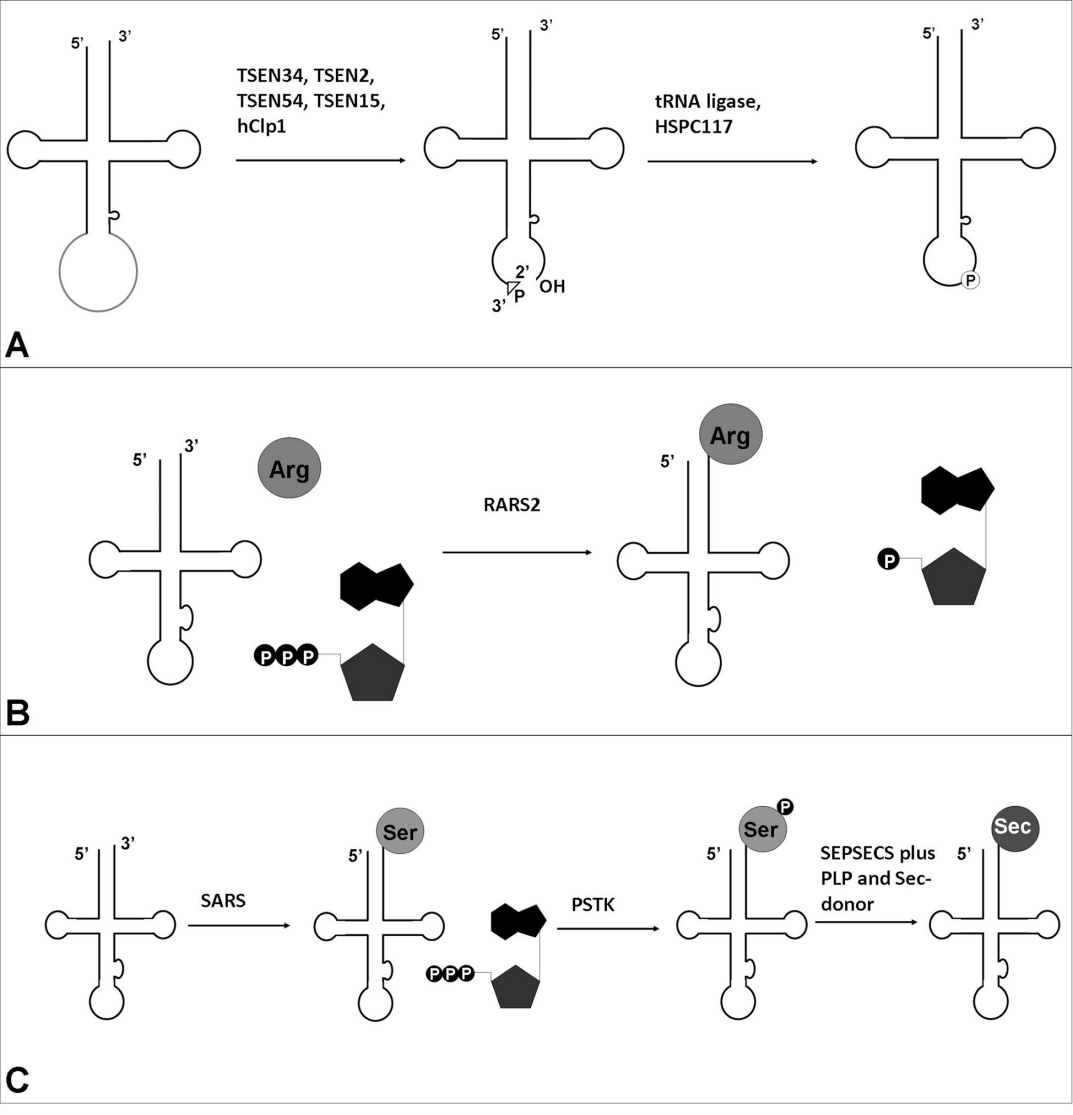
**Different RNA processing events in mammals**. **2A: **Eukaryotic splicing pathway of tRNA splicing in mammals. The TSEN complex is involved in the maturation of premature tRNAs and excises the tRNA into two halves; one 5'tRNA half with a 2'-3' cyclic phosphate at one exon-end and a 3'tRNA half with a 5'OH-group at the other exon-end. The final processing of tRNA maturation involves either direct ligation of the two tRNA halves by ligation through the Archaea-like pathway (by HSPC117, depicted) or indirect ligation through the yeast-like pathway (not depicted) [104]. Adapted from Calvin *et al*. [105]. **2B: **tRNA aminoacylation in mammals. RARS2 can bind to its cognate amino acid in an ATP dependent matter. This complex of ATP, RARS2 and arginine binds to the mt-tRNA-Arg and arginine will be transferred to its tRNA. Adapted from Antonellis *et al*. [83]. **2C: **Selenocysteine synthesis. Serine (Ser) is aminoacylated to tRNA-Sec by a seryl-tRNA synthetase (SARS). This Ser-tRNA-Sec complex is then converted by a kinase to a Sep-tRNA-Sec complex. In the presence of the cofactor pyridoxal phosphate (PLP) and the selenium donor selenophosphate (Se-donor) the SEPSECS enzyme converts the Sep-tRNA-Sec to Sec-tRNA-Sec. Adapted from Allmang *et al*. [87].

The tRNA splicing endonuclease (TSEN) complex is mutated in the majority of the PCH cases. The endonuclease complex is encoded by four different *TSEN *genes (*TSEN2, 15, 34, 54*) and consists of four protein subunits. Together they form one heterotetramic enzyme, consisting of two catalytic subunits (TSEN2 and TSEN34) and two structural subunits (TSEN54 and TSEN15) [72-74]. In mammals, maturation of tRNA necessitates removal of the 5' leader and 3' trailer sequences, addition of a CCA tail and various modifications [75]. Six percent of the human tRNA genes are intron-containing; this intron is not removed by the conventional splicing machinery, but by the TSEN complex. Only these intron-containing tRNAs require the TSEN complex for excising the tRNA into two halves; one 5'tRNA half with a 2'-3' cyclic phosphate at the cleavage site and a 3'tRNA half with a 5'OH-group at the other cleavage site (Figure 2A). The individual halves of the tRNA are ligated together again. Mature tRNAs are essential for translation of messengerRNA (mRNA) into proteins, as they transfer the correct amino acid to the ribosome to incorporate these amino acids at the growing peptide chain. Each amino acid has its own cognate tRNA and each tRNA has an anticodon sequence that can interact to the corresponding codon on mRNA sequence. In humans there are 506 different tRNA genes, meaning that there are more tRNA genes than codons. For certain tRNA species the majority of the tRNA genes are intron-containing: For example, 13 of the 14 tRNA-Tyr (GTA) genes contain an intron (Table 4). For other tRNAs there are no intron-containing tRNA genes at all: For example all tRNA-Gly (GCC, CCC, TCC) genes are not intron-containing. For further information on intron-containing and intronless tRNA genes, see [76].

**Table 4:** Overview of the number of human tRNA genes with an intron and the remaining corresponding tRNAs without an intron.

Human tRNA species with an intron (*anticodon*)	Number of human tRNA genes with an intron	Number of the remaining human tRNA genes without an intron (same anticodon)
Pro - *AGG*	1	9
Arg - *TCT*	5	1
Leu - *CAA*	5	2
Ile - *TAT*	5	0
Tyr - *ATA*	1	0
Tyr - *GTA*	13	1
Cys - *ACA*	1	0
Trp - *CCA*	1	8

Adapted from the Genomic tRNA database [76].

The TSEN complex is not only involved in tRNA splicing, it is also involved in mRNA 3'end formation. Knockdown of *TSEN2 *results in impaired mRNA 3'end formation of two housekeeping transcripts *GAPDH *and *EF1A *mRNA in HEK293 cells [73]. Furthermore the TSEN complex interacts with a lot of factors associated with the mRNA 3'end processing machinery [73,77].

In 2007, the human cleavage and polyadenylation factor I subunit (hClp1) was identified as siRNA kinase. hClp1 is a binding partner of the TSEN complex and its kinase activity is also active on 3'tRNA halves, furthermore hClp1 participates in mRNA 3'end formation [78-80]. It seems that multiple enzymes involved in RNA processing assemble into a molecular equivalent of a 'Swiss Army Knife', creating a complex that can cleave and process several different RNA species [79].

#### The TSEN complex and PCH

##### In vitro studies of the TSEN complex

In 2006 the yeast Sen complex was purified and different mutations in the complex were introduced; this showed that certain mutations abolished tRNA splicing activity whereas others do not affect tRNA splicing at all [74]. The authors also show that the same mutation can have a different effect on tRNA splicing, depending on the different tRNA: E.g. tRNA-Phe is usually not as strongly affected by mutations as tRNA-Tyr. Certain tRNA transcripts are perhaps spliced more readily than other tRNA transcripts [74]. This illustrates that it is difficult to predict how mutations in the TSEN complex will affect tRNA splicing in PCH patients. It is likely that tRNA splicing activity in patients is reduced, and not completely abolished as no patients to date have been described with two nonsense mutations in one of the *TSEN *subunits; this would abrogate tRNA splicing completely and lead to deprivation of certain amino acids, which would be non-compatible with life.

Generally, PCH4 patients have a null allele and a missense mutation in *TSEN54 *and are therefore more severely affected than PCH2 patients who carry two missense mutations in *TSEN54*. This genotype-phenotype correlation suggests that loss of TSEN function is the underlying disease mechanism in PCH2 and PCH4. One could hypothesize that a defective TSEN complex would lead to insufficient tRNA splicing, mainly affecting intron-rich tRNA genes, like tRNA-Tyr (Table 4). However Northern blot analysis of tRNA-Tyr from fibroblasts of three patients homozygous for the common mutation in *TSEN54 *did not show unspliced products. No significant difference was found in mature tRNA-Tyr levels in fibroblasts of patients and controls either [37]. To summarize, there is no evidence for a tRNA maturation defect in *TSEN*-mutated fibroblasts. Neuronal cells derived from patients would be a better substrate for tRNA maturation analysis, but unfortunately brain material is scarce and usually severely affected at the time of death. Induced pluripotent stem cell (iPS) technology might circumvent this problem, but even when patient fibroblasts can be converted to neuronal cells, there is no guarantee that these cells will show a phenotype. After all, not all neurons in a PCH case are affected.

It remains possible that other unknown (cell type specific) functions of the TSEN complex may play a role in the disease pathogenesis of PCH cases.

##### Zebrafish models of TSEN related PCH

Recently we established a zebrafish model for PCH. Knockdown of *tsen54 *by antisense morpholino injections in zebrafish embryos resulted in abnormalities in the mid-hindbrain and a developmental delay. The zebrafish embryos showed head hypoplasia and loss of structural integrity in the brain. The loss of structural definition in the brain is not due to a patterning defect, since fibroblast growth factor 8 (*Fgf8*) and orthodenticle homeobox 2 (*Otx2*), two developmental markers, were expressed in the correct regions. Instead the developing zebrafish embryo's showed increased levels of cell death, bearing comparisons to the neurodegeneration observed in PCH. This neurodegenerative phenotype is partially rescued by co-injecting human *TSEN54 *mRNA in zebrafish embryos [81].

Expression analysis of *tsen54 *in zebrafish shows a ubiquitous expression pattern, but higher expression in brain, primarily in the telencephalon and mid-hindbrain boundary. This is in line with the expression of human *TSEN54 *mRNA at eight weeks of gestation, in which high expression in the developing telencephalon and metencephalon is observed [81]. The human cerebellum begins its development at six weeks of gestation and continues growing into the postnatal period [82]. With careful monitoring in PCH4/PCH5 cases, one can already measure a decline in transverse cerebellar diameter at 16 weeks of gestation, indicating a very early onset of the neurodegeneration [11].

Antisense morpholino effects diminish after a few days post-injection. Therefore we also developed a stable *tsen54 *knockout zebrafish carrying a premature stop codon. When bred to homozygosity these zebrafish mutants were viable at 9 days post fertilization (dpf). The absence of a major brain phenotype and survival up to 9 --dpf may be explained by the presence of maternal *tsen54 *during embryogenesis [81]. Redundancy of the protein in zebrafish is not likely, as eventually these mutant zebrafish die after 9 days.

#### RARS2 and PCH

RARS2, mutated in PCH6 and PCH1, is one of the 36 human tRNA synthetases. By the usage of ATP, the tRNA synthetase binds to its cognate amino acid. This complex of ATP, tRNA synthetase and amino acid, binds to the appropriate tRNA whereto the amino acid will be transferred (Figure 2B) [83]. RARS2 is involved in the aminoacylation of arginine (Arg) to its mitochondrial- tRNA-Arg. In *RARS2 *mutated (c.110+5A > G) fibroblasts, a reduction in the amount of the mt- tRNA-Arg was observed. Despite this reduction, the residual mt- tRNA-Arg transcript was almost completely acylated, suggesting that uncharged mt-tRNA-Arg transcripts are unstable [12]. Morpholino-directed knockdown of *rars2 *in zebrafish resulted in a similar neurodegenerative phenotype as *tsen54 *knockdown, suggesting the same pathogenesis for PCH2/4 and PCH6, although it is still not understood how RARS2 is involved in the development of the pons and cerebellum [81].

Alternative functions of RARS2 compromising the same biological pathway as the TSEN complex could also be the underlying mechanism in PCH [73,78,81]. For some of the tRNA synthetases alternative functions are known in processes like conventional splicing, apoptosis, viral assembly, regulation of transcriptional and translational processes and angiogenic signaling [83-85].

#### SEPSECS and PCCA

Mutations in *SEPSECS *are associated with PCCA (progressive cerebello-cerebral atrophy). The SEPSECS enzyme (Figure 2C) is involved in the final step of the selenocysteine (Sec) synthesis [86,87]. Selenocysteine lacks its own tRNA synthetase and in contrast to the other amino acids selenocysteine is synthesized on its cognate tRNA. The codon for selenocysteine is UGA, normally encoding for translation termination, however depending on the flanking sequences of the UGA, this codon is recoded for a selenocysteine. Prior to selenocysteine synthesis, serine (Ser) is aminoacylated to tRNA-Sec by a seryl-tRNA synthetase; this Ser-tRNA-Sec complex is then converted by a kinase to a Sep-tRNA-Sec complex. As a final step SEPSECS converts the Sep-tRNA-Sec to Sec-tRNA-Sec. It is not likely that *SEPSECS *missense mutations (p.A239T, p.Y334K) completely abolish its function, as deprivation of selenocysteins is associated with lethality in mice [88].

### Discussion

The identification of mutations in genes involved in transcription and translation in neurological disorders shows that these processes are important in (developing) neurons [83,89-94]. The TSEN complex, RARS2 and SEPSECS proteins, all share involvement in essential processes in protein synthesis and mutations in the corresponding genes all lead to a severe phenotype of pontocerebellar hypoplasia, often in combination with cortical involvement. One explanation could be that developing neuronal tissue is sensitive to dysregulation of protein synthesis [83,92-94]. This hypothesis is supported by mice with a homozygous missense mutation in the alanyl-tRNA synthetase gene (*AARS*). This mouse has increased levels of mischarged tRNAs which lead to intracellular accumulation of misfolded proteins in neurons, this induces the unfolded protein response (UPR) and these mice develop a neurodegenerative phenotype, with Purkinje cell death and subsequent ataxia [95]. However UPR activation has not been detected in postmortem brain of PCH2 patients carrying a *TSEN *mutation, but one can argue that UPR activation is an early event in the pathogenesis of PCH and that postmortem studies do not capture this event [19].

On the other hand it is possible that there is a time frame during embryogenesis in which there is an extra high demand for protein synthesis in neuronal tissue in the early post-migratory stage. Human brain tissue expresses relatively high overall levels of nuclear and mitochondrial encoded tRNAs, which might be due to higher levels of translation in the CNS compared to other tissue [96]. Moreover, malnutrition in rats during the prenatal period has severe consequences for brain development and the cerebellum in particular [97]. Protein malnutrition both prenatally and postnatally, results in reduced brain weight, thinner cerebral and cerebellar cortices, reduced numbers of neurons, deficient myelination and reduced dendritic spines of cortical neurons [98,99]. Therefore it is very likely that nutrients and proteins are highly essential for normal brain development.

Why mutations in other genes involved in protein synthesis lead to completely different phenotypes remains unclear. For example, mutations in other genes involved in tRNA charging like *KARS*, *YARS *and *GARS *lead to different types of Charcot-Marie-Tooth neuropathy [83,100,101]. Mitochondrial aspartyl-tRNA synthetase (*DARS2*) mutations cause leukoencephalopathy with brain stem and spinal cord involvement and lactate elevation (LBSL). Patients with LBSL have profound white matter abnormalities in their cerebrum, pons and spinal cord. There are some similarities between LBSL and PCH, like cerebellar involvement and spasticity, but the differences are more evident. Patients have slowly progressive cerebellar ataxia, dorsal column dysfunction, occasionally a mild cognitive decline and the age of onset is usually during childhood or even later. It is unclear how mutations in *DARS2 *lead to LBSL [89].

Not all mutations in tRNA synthetase genes result in a neurological phenotype. Missense mutations in the mitochondrial Seryl-tRNA Synthetase gene (*SARS2*) give rise to a multi-organ disease with Hyperuricemia, Pulmonary Hypertension, Renal Failure and Alkalosis (HUPRA-syndrome). Although there is failure to thrive and a global developmental delay, patients exhibit no neurological symptoms and brain ultrasound was reported to be normal [102]. Compound heterozygote mutations in mitochondrial histidyl-tRNA synthetase gene (*HARS2*) were found in one family with ovarian dysgenesis and progressive sensorineural hearing loss (Perrault syndrome) [103].

In summary, the most likely explanation for the neurological phenotype in the disorders described above is that maturing neurons are more vulnerable for defects in protein synthesis than other tissues, but this does not explain the difference between disease presentation shared by mutations of *TSEN, RARS2 *and *SEPSECS*, in which the cerebellum appears to be preferentially affected and other diseases *(KARS, YARS, GARS, SARS2)*, with a different disease presentation.

## Conclusions and Outlook

During the last decade many genes involved in protein synthesis have been associated with different neurological diseases. Several genes of PCH subtypes have been described, in view of the shared function in tRNA processing, a defect in protein synthesis seems the most likely pathomechanism in PCH. Hopefully identification of new genes in PCH subtypes will provide further insight and lead us to a common disease pathway. Also further research on TSEN and/or RARS2 function in PCH models is necessary to elucidate the question why solely the brain, and specifically the cerebellum and pons are affected in PCH.

## Competing interests

The authors declare that they have no competing interests.

## Authors' contributions

All authors made substantial contributions and have given final approval to the version being published.
